# Short-term DAPT after coronary stenting has similar ischemic and bleeding outcomes as long-term DAPT: a 5-year population-based cohort study

**DOI:** 10.1007/s11845-022-03171-y

**Published:** 2022-09-29

**Authors:** Sriraag Balaji Srinivasan, Amro Sehly, Biyanka Jaltotage, Simon Qin, Abdul Rahman Ihdayhid, James Marangou, James M. Rankin, Frank M. Sanfilippo, Girish Dwivedi

**Affiliations:** 1 Department of Cardiology, Fiona Stanley Hospital, Perth, WA Australia; 2 School of Medicine, University of Western Australia, Perth, WA Australia; 3 School of Population and Global Health, University of Western Australia, Perth, WA Australia; 4 Harry Perkins Institute of Medical Research, Perth, WA Australia; 5 Medical School, Curtin University, Perth, WA Australia; 6 Menzies School of Health Research, Darwin, NT Australia

**Keywords:** Bleeding, DAPT, Stenting, Thrombosis

## Abstract

**Background:**

Optimal duration of dual antiplatelet therapy (DAPT) following percutaneous coronary intervention (PCI) remains controversial.

**Aim:**

We investigated the relationship between DAPT duration following PCI and long-term ischemic and bleeding outcomes under real-world conditions.

**Methods:**

Patients aged ≥ 65 years who underwent PCI with stenting in Western Australian hospitals between 2003 and 2008 and survived 2 years were identified from linked hospital admissions data. The primary outcome was major adverse cardiovascular and cerebrovascular events (MACCE) defined as a composite of all-cause death and admissions for acute coronary syndrome (ACS), coronary artery revascularization procedure, stroke, and major bleeding. Secondary outcomes were ACS admissions, all-cause death, and major bleeding admissions. Patients were followed up for 5 years from initial PCI.

**Results:**

A total of 3963 patients were included in the final analysis. The mean age of the cohort was 74.5 ± 6.1 years with 67.3% males. No significant difference was seen with 6–12, 12–18, or 18–24 months DAPT, compared to 0–6 months DAPT duration for MACCE and all secondary outcomes at 3- and 5-year post-PCI.

**Conclusion:**

There is no significant difference in both bleeding and ischemic outcomes in long-term DAPT as compared to short-term DAPT for first- and second-generation drug-eluting stents in a real-world population.

## Introduction

Optimal duration of dual antiplatelet therapy (DAPT) following percutaneous coronary intervention (PCI) with stent insertion remains controversial. Randomized trials have demonstrated that long-term DAPT beyond 1 year results in fewer ischemic events at the expense of greater bleeding events [1, 2]. As such, individualized ischemic and bleeding risks are considered when selecting optimal DAPT duration [3]. However, trial populations are not representative of real-world patients, and their findings should therefore be met with caution. A further layer of complexity is added when developments in stent technology and changes in practice patterns over time are considered; these factors are challenging to capture in rigid protocol-driven clinical trials. Additionally, most trials have shorter follow-up durations, offering limited long-term outcome data. Population-based studies provide large, long-term, real-world data that can address these issues. We investigated the relationship between DAPT duration following PCI and ischemic and bleeding outcomes under real-world conditions.

## Methods

Patients aged ≥ 65 years who had a PCI with stent implantation for any indication in Western Australian (WA) hospitals between 2003 and 2008 and survived 2 years following PCI were identified from linked hospital admissions data. Pharmaceutical Benefits Scheme (PBS) data was used to identify DAPT duration. The PBS is a national Australian Government program that subsidizes the cost of prescription medicine. Demographic and outcome data was obtained from Western Australian Hospital Morbidity Data Collection, Emergency Department Data Collection and Mortality Register databases. Patients were followed up for 3 years (a total of 5 years from initial PCI). The primary outcome of the study was major adverse cardiovascular and cerebrovascular events (MACCE) defined as a composite of all-cause death and admissions for acute coronary syndrome (ACS), coronary artery revascularisation procedure, stroke and major bleeding. Secondary outcomes were ACS admissions, all-cause death, and major bleeding admissions. Patients were stratified into four DAPT duration groups at 6-month intervals, 0–6 months, 6–12 months, 12–18 months and 18–24 months. Adjusted Cox regression analysis was done on outcome data at 3-years post-PCI and 5-years post-PCI, comparing DAPT duration outcomes to the baseline of 0–6 months. Outcome analysis was adjusted based on patient, lesion, and procedure related factors.

## Results

A total of 7407 patients underwent coronary stent implantation in WA hospitals between 2003 and 2008, of which 6466 survived 2 years following PCI. A further 2503 were excluded due to unavailable medication records, with a final total of 3963 included in the analysis. The mean age of the cohort was 74.5 ± 6.1 years with 67.3% males. More than half of patients underwent PCI for acute coronary syndrome (53.4%), 19.8% unstable angina, 17.8% non-ST elevation myocardial infarction, and 15.8% ST elevation myocardial infarction. The remaining patients had stable angina (46.6%). A total of 56.4% of patients had single-stent intervention, with the remainder (43.6%) having multiple stents inserted. Drug-eluting stents (DES) comprised 84.1% of all stents used; the other 15.6% was bare metal stents (BMS). All patients were dispensed aspirin and clopidogrel as DAPT. A total of 1158 (29.2%) received DAPT for 0–6 months, 1352 (34.1%) received DAPT for 6–12 months, 1034 (26.1%) received DAPT for 12–18 months, and 419 (10.6%) received DAPT for 18–24 months.

**Fig. 1:**
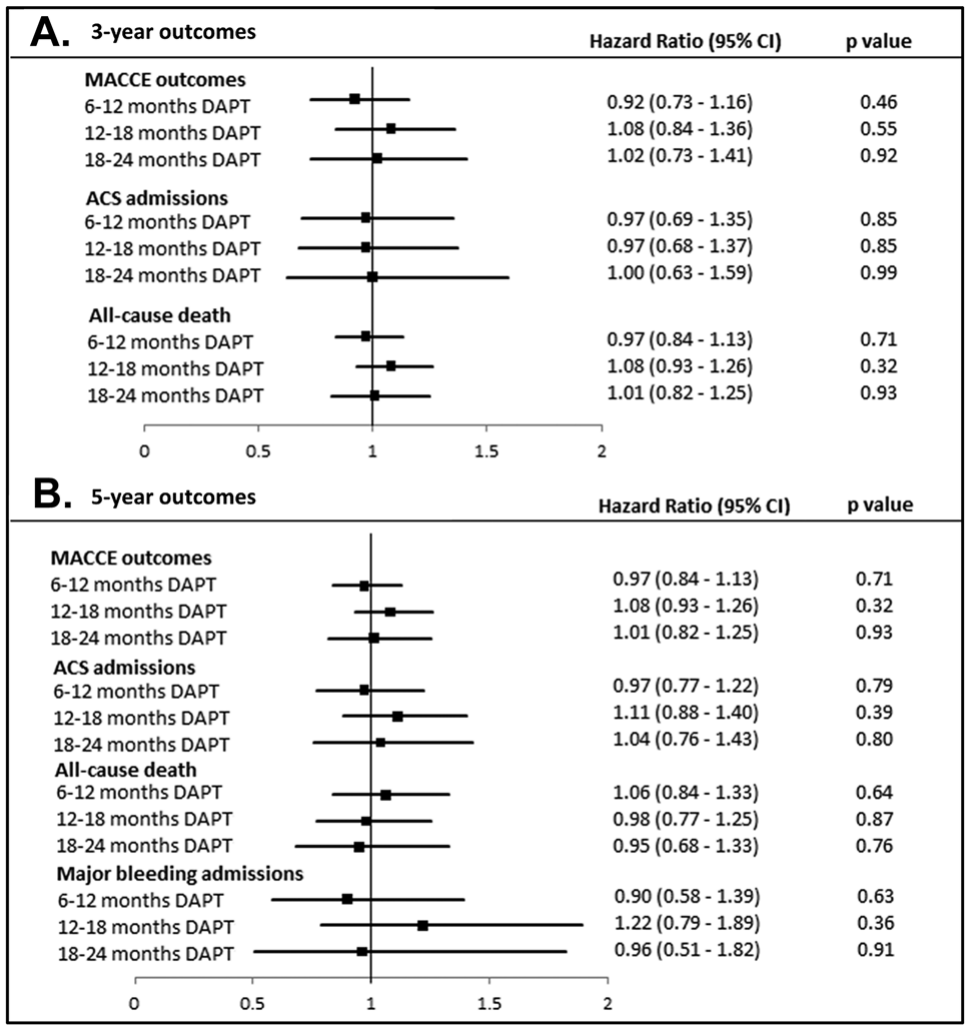
Results from adjusted Cox regression analyses at 3-years (**A**) and 5-years (**B**) post-coronary stent implantation. Hazard ratios (HRs) are with reference to outcomes for 0- to 6-month DAPT. Central boxes represent HRs, and error bars represent 95% confidence intervals. *DAPT* dual antiplatelet therapy, *ACS* acute coronary syndrome, *MACCE* major adverse cardiovascular and cerebrovascular events

No significant difference was seen with 6–12 months, 12–18 months, or 18–24 months DAPT, compared to 0–6 months DAPT duration for MACCE at 3-year and 5-year post-PCI. No significant difference was seen in ACS admission, all-cause death, and major bleeding outcomes at 3-year and 5-year post-PCI for these groups as compared to 0- to 6-month DAPT duration (Fig. 1).

## Discussion

For over a decade, multiple randomised trials have studied the optimal duration of DAPT, yet the answer remains elusive. The landmark DAPT trial established the outcome benefits of long-term DAPT beyond 12 months, with a notable increase in major bleeding admissions [1]. Conversely, the ITALIC trial found no difference between short-term and long-term DAPT, demonstrating no change in thrombotic and bleeding risks in 6 months vs 24 months of DAPT [4]. Meta-analyses have also demonstrated differing results, with more recent data trending towards shorter duration DAPT [5]. These trials lack real-world data and long-term follow-up, a distinct strength of population-based studies.

Our study demonstrated that there was no significant difference in both bleeding and thrombotic outcomes in long-term DAPT as compared to short-term DAPT with predominantly first- and second-generation DES in a real-world population. With the established improvement in newer generation DES in thrombotic risk, our findings align with other work suggesting low thrombotic risk with short-term DAPT [6]. Conversely, similar bleeding risks provide reassurance in patients requiring prolonged DAPT. This is encouraging in the era of personalized medicine, permitting flexibility in DAPT duration depending on individual thrombotic and bleeding risk. The limitations of our study include its retrospective nature, the age of the data, the use of clopidogrel in contrast to ticagrelor, the high percentage of BMS use, and inherent limitations of database data.
